# Molecular portrait of breast cancer in China reveals comprehensive transcriptomic likeness to Caucasian breast cancer and low prevalence of luminal A subtype

**DOI:** 10.1002/cam4.442

**Published:** 2015-03-18

**Authors:** Xiaoyan Huang, Matteo Dugo, Maurizio Callari, Marco Sandri, Loris De Cecco, Barbara Valeri, Maria Luisa Carcangiu, Jingyan Xue, Rui Bi, Silvia Veneroni, Maria Grazia Daidone, Sylvie Ménard, Elda Tagliabue, Zhimin Shao, Jiong Wu, Rosaria Orlandi

**Affiliations:** 1 Department of Breast Surgery, Fudan University Shanghai Cancer Center and Department of Oncology, Shanghai Medical College, Fudan UniversityShanghai, 200032, China; 2 Functional Genomics and Bioinformatics, Department of Experimental Oncology and Molecular Medicine, Fondazione IRCCS Istituto Nazionale dei TumoriMilan, Italy; 3 Biomarkers Unit, Department of Experimental Oncology and Molecular Medicine, Fondazione IRCCS Istituto Nazionale dei TumoriMilan, Italy; 4 Molecular Targeting Unit, Department of Experimental Oncology and Molecular Medicine, Fondazione IRCCS Istituto Nazionale dei TumoriMilan, Italy; 5 Department of Pathology, Fondazione IRCCS Istituto Nazionale dei TumoriMilan, Italy; 6 Department of Pathology, Fudan University Shanghai Cancer Center, Shanghai Medical College, Fudan UniversityShanghai, 200032, China

**Keywords:** Breast cancer, Chinese/Caucasian, gene expression profiling, miRNA profiling, race/ethnicity

## Abstract

The recent dramatic increase in breast cancer incidence across China with progressive urbanization and economic development has signaled the urgent need for molecular and clinical detailing of breast cancer in the Chinese population. Our analyses of a unique transethnic collection of breast cancer frozen specimens from Shanghai Fudan Cancer Center (Chinese Han) profiled simultaneously with an analogous Caucasian Italian series revealed consistent transcriptomic data lacking in batch effects. The prevalence of Luminal A subtype was significantly lower in Chinese series, impacting the overall prevalence of estrogen receptor (ER)-positive disease in a large cohort of Chinese/Caucasian patients. Unsupervised and supervised comparison of gene and microRNA (miRNA) profiles of Chinese and Caucasian samples revealed extensive similarity in the comprehensive taxonomy of transcriptional elements regulating breast cancer biology. Partition of gene expression data using gene lists relevant to breast cancer as “intrinsic” and “extracellular matrix” genes identified Chinese and Caucasian subgroups with equivalent global gene and miRNA profiles. These findings indicate that in the Chinese and Caucasian groups, breast neoplasia and the surrounding stromal characteristics undergo the same differentiation and molecular processes. Transcriptional similarity across transethnic cohorts may simplify translational medicine approaches and clinical management of breast cancer patients worldwide.

## Introduction

Breast cancer is a leading cause of morbidity and mortality among female cancer patients worldwide. The disease is categorized according to at least three distinct molecular and clinical subtypes with dramatically different outcome and response to therapeutic agents 1,2. The luminal subtype (estrogen receptor [ER]-positive) is the most common and heterogeneous group and is treated with endocrine therapy. The human epidermal growth factor receptor 2 (HER2 or ERBB2)-amplified subtype has recently been successfully treated with anti-HER2 targeted approaches. The triple-negative breast cancer (TNBC) subgroup, lacking expression of hormonal receptors and HER2, has an unfavorable prognosis and is currently treated after surgery with standard chemotherapy due to its significant chemosensitivity. The incidence of TNBCs, also known as basal-like breast tumors, is increased in patients with germline Breast Cancer 1, Early Onset (BRCA1) mutations 2 and in premenopausal women of African ancestry 3. Heterogeneity and complexity of breast cancer have been extensively detailed by large genomic and transcriptomic studies 4,5 that must be translated into a personalized management of the disease based on genetic and molecular traits of each breast tumor, but also accounting for social and environmental conditions of each patient. In this context, it remains controversial whether racial/ethnic information can help to explain molecular features, etiological diversity and progression of breast cancer. Although there is abundant epidemiological evidence that race/ethnicity is associated with disparities in cancer incidence and mortality 6, and some studies have shown that such differences are affected by social–economic conditions leading to inequality in access to effective medical care or exposure to risk factors 6,7, it is still unclear whether race/ethnic variability in cancer also reflects differences in cancer biology.

Compared with women in Western countries, Asian women still have lower incidence rates of breast cancer and lower median age at the diagnosis (48–50 years) 8; however, the disease has increased dramatically over the past 30 years in urban areas of China and other Asian developing countries. Fan et al. 9 highlighted how socioeconomic development, accelerated urbanization, higher lifetime expectancy, and the aging of the Shanghai population have greatly contributed to the rapid change in risk factors and the increase in breast cancer incidence and mortality. Since this increase is likely to continue in the next decades across all of China 8, there is an urgent need for molecular and clinical detailing of breast cancer in this population. Epidemiological and genomic differences between Asian and Caucasian breast cancer have been reported based on data from independent analyses of Asian and Caucasian series 10,11 and from heterogeneous cohorts of Asian/Pacific Islander women 12,13, but no studies have been conducted to compare Chinese and Caucasian samples simultaneously, even though studies of breast cancer aggressiveness in Afro-American women have underscored the great advantage of simultaneous analysis in large cohorts of women of African or Caucasian ancestry in the US 3,7. Herein, we investigated biological diversity among breast cancers from Chinese and Caucasian women by comparing comprehensive gene and microRNA (miRNA) profiles of samples analyzed simultaneously and derived from a unique transethnic cohort of Chinese Han breast cancer patients surgically resected at Fudan Cancer Center of Shanghai (China) and of Caucasian Italian patients surgically resected at Fondazione IRCCS Istituto Nazionale dei Tumori of Milan (INT, Italy). Chinese and Caucasian comprehensive transcriptomic data were examined for patterns of similarity and dissimilarities using unsupervised and supervised analysis, and breast cancer molecular taxonomy was explored and compared in both Chinese and Caucasian gene profiles.

## Materials and Methods

### Samples and clinical data

Tissue samples from 78 Chinese Han and 97 Caucasian Italian consecutive primary breast tumors were collected for gene and miRNA profiling at Breast Surgery Units of Cancer Hospital-Fudan University of Shanghai and INT of Milan in 2007. Specimens were frozen in liquid nitrogen within 1 h in the surgical room at Shanghai and within 4 h in the Pathological Department at Milan, after which all samples were kept at −80°C in the Italian Center. Bilateral synchronous breast tumors, that were 2% and 3% in Chinese and Caucasian series, respectively, were excluded from the profiled cohort. Clinical–pathological data on consecutive breast cancer from 1057 Chinese and 1047 Italian patients were collected in years 2005 and 2006 in the same Institutions. The study was approved by the Medical Ethics Committee of both Institutions. All data and tissue samples were obtained upon informed consent and according to respective institutional rules.

### Immunohistochemical analysis

ER status was determined by immunohistochemistry using mouse monoclonal anti-human ER antibody (Dako, Carpinteria, CA, clone 1D5) on Chinese tumors or using ER-ICA (estrogen receptor immunocytochemical assay) assay on Italian tumors (Abbott, Abbott Park, IL) according to the manufacturer’s recommendations. Tumors were considered receptor-positive if more than 1% of malignant cells showed nuclear staining. To assess agreement of ER status determination between the two hospitals, independent pathologists in Milan and Shanghai reviewed a random sample of 100 breast cancer cases and found a Cohen’s κ statistic of 0.94, indicating substantial agreement beyond chance and an overall concordance of 98%.

Subtyping of breast cancer samples according to immunohistochemical (IHC) determination of their hormonal and HER2 receptors was based on Carey classification 3: Luminal HER2− (ER+ and/or PR+, HER2−); Luminal HER2+ (ER+ and/or PR+, HER2+); HER2+/ER− (ER−, PR−, HER2+) and ER−/PR−HER2−. HER2/neu status was determined using polyclonal rabbit anti-Human c-erbB-2 antibody (Dako) in both Chinese and Italian tumors. Staining was quantified using a score of 0, 1, 2 and 3; tumors with a score of 3 were considered HER2 positive.

### RNA extraction

Total RNA was extracted using the miRNeasy Mini Kit (Qiagen, Valencia, CA) at INT from 50 to 100 mg of each Chinese and Italian tissue sample homogenized using a bench-top homogenizer (MM200, Retsch, Germany) in 1 mL of TRIzol reagent (Invitrogen, Life Technologies, Grand Island, NY). An aliquot of 1–2 *μ*g of total RNA was retained for miRNA analysis, while the remaining material was cleaned-up and treated with RNAse-free DNAse to remove genomic DNA traces, using the RNeasy Mini Kit (Qiagen) according to the manufacturer’s instructions. Qualitative analysis of RNA was performed using Agilent RNA 6000 Nano kit and Agilent 2100 Bioanalyzer.

### Gene and miRNA profiling

RNA samples were processed for gene and miRNA profiling on the Illumina platform at the Functional Genomics Facility of INT. For gene profiling analysis, 800 ng of total RNA was reverse transcribed, biotin-labeled, and amplified using Illumina RNA TotalPrep Amplification kit (Ambion, Life Technologies, Grand Island, NY) as per the manufacturer’s instructions. One *μ*g of each cRNA amplified sample was added to Hyb E1 hybridization buffer containing 37.5% (w/w) formamide and hybridized to array HumanHT-12-v3 expression Bead Chip (Illumina, Inc., San Diego, CA) at 58°C for 18 h. Array chips were washed and stained using 1 *μ*g/mL of Cy3-streptavidin (Amersham Biosciences). For miRNA profiling, miRNAs were amplified with the Illumina human_v2 MicroRNA expression profiling kit, based on the DASL (cDNA-mediated Annealing, Selection, Extension, and Ligation) assay according to the manufacturer’s instructions. Briefly, 400 ng/sample of total RNA was converted to cDNA. After annealing with miRNA-specific oligo pools, PCR amplification and fluorescent labeling, probes were hybridized on Illumina miRNA BeadChips, allowing analysis of 1146 sequences covering 97% of miRNAs present in the miRBase v12.0. After hybridization, fluorescent signals were detected by the Illumina BeadArray^TM^ reader.

### Microarray data preprocessing

Raw expression data were collected from scanned images using Illumina BeadStudio v3.3.8 (Illumina, Inc.) and processed using the *lumi* package 14 from Bioconductor v2.11 15. Probes from gene expression data were reannotated using the *illuminaHumanv3.db* package v1.16.0. Quality control was performed on raw and processed data by evaluation of array intensity distributions, distances between arrays, and principal component analysis (PCA) for the identification of outliers. All samples passed quality-control procedures. Gene and miRNA raw data were log2-transformed, normalized with Robust Spline Normalization and filtered, keeping only the probes with a detection *P* < 0.01 in at least one sample. Multiple probes representing the same gene were collapsed and the probe with the highest detection rate, that is, the percentage of samples in which the probe had a detection *P* < 0.01, was selected. In the case of equal detection rates, the most variant probe according to interquartile range (IQR) was selected. Final data included 15,929 unique genes and 848 unique miRNAs. Expression data were deposited in the Gene Expression Omnibus data repository (GEO) with accession number GSE59595. Gene expression and microRNA raw data from Buffa et al. 16 were used as independent datasets in the comparison of Chinese and Caucasian Italian profiles and were downloaded from GEO (accession numbers GSE22219 and GSE22216, respectively) and subjected to the above preprocessing procedure. Common features in GSE22219 (Illumina human Ref-8 v1.0) and Chinese-Italian datasets were selected by gene symbol.

Gene expression data from 81 Taiwanese Chinese Han patients (GSE48390 17) and from 100 women of National University Hospital of Singapore (GSE36772) were downloaded from GEO and used to validate the lower prevalence of luminal A tumors in Chinese women. Both cohorts were profiled on Affymetrix platform. Raw CEL files were processed using the frozen robust multiarray analysis method 18. Multiple probes representing the same gene were collapsed selecting the probe with the highest average expression.

### Unsupervised analysis

Groups of correlated genes and miRNAs were identified in Chinese and Caucasian Italian datasets separately, using the approach described by Callari et al. 19. Briefly, genes with IQR > 0.4 from the Chinese dataset were partitioned using hierarchical clustering with agglomerative average linkage as linkage criterion and 1- the Pearson’s correlation coefficient as a distance measure. The resulting dendrogram was cut at a correlation value of 0.6 and clusters containing at least 10 genes were selected. Such thresholds were selected in order to consider sufficiently large groups of genes with biologically meaningful correlations, ensuring representation of pivotal breast cancer gene-clusters such as “ER” and “proliferation-related.” A dendrogram was then generated for each cluster using Caucasian Italian gene expression data and the same correlation thresholds. A cluster was considered validated if at least three genes had a correlation >0.6 in the Italian dataset. The same approach was applied to Chinese miRNA data, but in view of the different number of features and data characteristics, the correlation threshold was set at 0.4; clusters containing at least five miRNAs were selected and validated on the Caucasian Italian dataset if at least three miRNAs passed the correlation threshold. The same procedure was repeated in the reverse order, starting from the Caucasian Italian gene and miRNA datasets and validating the clusters in the corresponding Chinese datasets or starting from the GSE22219 and GSE22216 datasets and validating selected clusters in both Chinese and Caucasian Italian data. Overrepresentation of gene lists relevant to breast cancer 1,20–22 was analyzed using the hypergeometric test, in which *P* < 0.01 was considered significant.

### Sample classification according to breast cancer molecular signatures

Samples were classified as ER-positive and ERBB2-positive by setting the expression value thresholds corresponding to the local minima of the bimodal density distribution of the each gene, calculated using the *turnpoints* function of the *pastecs* package 23. Hierarchical clustering with average linkage and Euclidean distance was applied to classify samples into breast intrinsic molecular subtypes, using the PAM50 gene signature 24 or to assign samples to the claudin-low subtype using the gene list of Prat et al. 25. Five PAM50 genes (*NUF2*, *CXXC5*, *MIA*, *ORC6L*, and *MYBL2*) were not present on the platform or were filtered out as never detected.

Identification and stability analysis of extracellular matrix (ECM) clusters was performed using the Large Average Submatricies (LAS) biclustering algorithm and the statistical methods described in Triulzi et al. 26. A list of genes including *CTSS*, *GZMK*, *MMP7*, *MMP9*, *SELL*, *SPOCK2*, and *VCAM1* 21 and the proposed ECM3 signature 26 were used to identify ECM1 and ECM3 clusters, respectively. Similarity between the intrinsic and ECM subtypes identified in the two datasets was assessed by subclass mapping using the SubMap module of GenePattern software v3.8 27. In addition, PCA was applied on global miRNA data using the first two principal components to compare the distributions of tumors in different subtypes.

### Statistical analysis of gene and miRNA expression data

Differentially expressed genes in the Chinese and Italian samples or among biological subgroups were identified by linear modeling as implemented in the *limma* package 28, using ethnicity and molecular subtype as covariates. Multiple-testing correction was performed using the Benjamini–Hochberg false discovery rate (FDR). Genes and miRNAs with FDR < 0.05 and absolute fold-change ≥2 between Chinese and Italian samples or in at least one of the possible pairwise comparisons between subtypes were considered significantly differentially expressed. Association between expression data and storage time was assessed through linear modeling using the *lm* function in R. Significance threshold was set at nominal *P* < 0.001. Breast cancer signatures were downloaded from the GeneSigDB database 29 using the keywords “human” and “breast.”

### Statistical analysis of clinic-pathological variables

Association between categorical variables and ethnicity was assessed using the chi-squared test or Fisher’s exact test. Partial correlation was used to estimate the degree of association between ER status and age adjusted for the effect of a set of controlling variables (tumor size, grade, nodal status, ER−/PR−/HER2− vs. HER2+/ER− vs. Luminal HER2− vs. Luminal HER2+). Significance threshold was set at *P* < 0.05.

## Results

### Clinico-pathological features of Chinese/Caucasian cohort

Table1 lists the clinico-pathological characteristics of the profiled breast tumors from the consecutive cohort of Fudan Cancer Center of Shanghai and INT of Milan. Age of disease onset differed significantly in the two groups, with median ages of 50 and 60 years in the Chinese and Caucasian series, respectively, and with 47% of Chinese versus 68% of Caucasian women older than 50 years. Although not statistically significant, the percentage of ER-positive patients as assessed immunohistochemically was lower in the Chinese group (67% vs. 77%), according to Fan et al. 9, with a similar trend evidenced when ER status was assessed based on gene expression. Other clinico-pathological features such as histological grade, size and lymph nodal status were not significantly associated with race/ethnicity. Comparison of 1057 Chinese and 1047 Caucasian consecutive breast cancer patients from the same Institutions (Table2) to verify the prevalence of ER-positive disease in a larger cohort revealed a significant disparity between Chinese and Caucasian women in both age of incidence and ER status; 54% of women in the Chinese series were older than 50 years versus 69% of Caucasians (median age 51 and 59, respectively) and 63% of Chinese versus 85% of Caucasian tumors were ER-positive.

**Table 1:** Clinical, pathological, and molecular characteristics of 78 Chinese and 97 Caucasian primary consecutive breast cancer patients surgically treated at Cancer Hospital-Fudan University of Shanghai and INT of Milan, respectively, and analyzed for gene and miRNA expression

	Chinese series (%)	Caucasian series (%)	*P*-value
Number of patients	78 (100)	97 (100)	
Age (years)			
Median	50	60	0.009
Range	32–78	35–85	
≤50	41 (53)	31 (32)	
>50	37 (47)	66 (68)	
Size (cm)			
Median	2.5	2.0	0.124
Range	1.0–7.0	0.4–11.0	
≤2.0	47 (60)	45 (46)	
>2.0	31 (40)	50 (52)	
NA	0 (0)	2 (2)	
Histological grade			
I + II	40 (51)	46 (47)	0.195
III	26 (33)	48 (49)	
NA	12 (16)	3 (4)	
Lymph node			
Positive	35 (45)	46 (47)	0.498
Negative	36 (46)	36 (37)	
NA	7 (9)	15 (16)	
ER status (IHC)			
Positive	52 (67)	75 (77)	0.162
Negative	26 (33)	22 (23)	
ER status (gene)			
Positive	45 (58)	69 (71)	0.090
Negative	33 (42)	28 (29)	
HER2 status (IHC)			
Positive	10 (13)	10 (10)	0.780
Negative	68 (87)	87 (90)	
HER2 status (gene)			
Positive	12 (15)	11 (11)	0.574
Negative	66 (85)	86 (89)	
IHC subtypes			
ER−/PG−/HER2−	17 (22)	13 (13)	0.218
HER2+/ER−	6 (8)	4 (4)	
Luminal HER2−	51 (65)	70 (72)	
Luminal HER2+	4 (5)	10 (10)	

ER, estrogen receptor; NA, not available; IHC, immunohistochemistry; HER2, human epidermal growth factor receptor 2.

**Table 2:** Age and ER status of 1057 and 1047 primary consecutive breast cancer patients surgically treated in the years 2005–2006 at Cancer Hospital-Fudan University of Shanghai and INT of Milan, respectively

	Chinese series (%)	Caucasian series (%)	*P*-value
Number of patients	1057 (100)	1047 (100)	
Age (years)			
Median	51	59	<0.001
Range	23–85	25–92	
≤50	487 (46)	324 (31)	
>50	570 (54)	723 (69)	
ER status			
Positive	663 (63)	885 (85)	<0.001
Negative	394 (37)	162 (15)	

ER (estrogen receptor) status was assessed by immunohistochemistry.

Age at onset was significantly associated with ER status in both Chinese (adjusted mean age of 49.4 years in ER-negative and 53.3 years in ER-positive, *P* = 0.0049) and Caucasian series (adjusted mean age of 51.4 years in ER-negative and 57.1 years in ER-positive, *P* = 0.046).

### Comparison of global gene and miRNA expression profiles of Chinese and Caucasian samples

**Figure 1:**
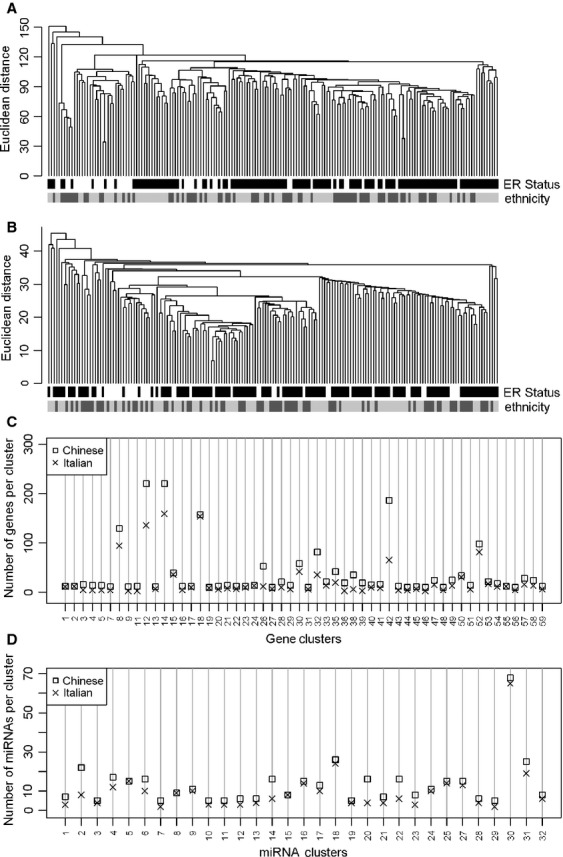
Global transcriptional similarity of Chinese and Caucasian samples. Dendrogram, obtained by hierarchical clustering of gene expression (A) and microRNA (miRNA) (B) data of the Chinese–Caucasian cohort, separated samples independently of race/ethnicity. The color bar at the bottom of the dendrogram shows hormone receptor status (assessed by immunohistochemistry) and race/ethnicity. Global genes and miRNA from the Chinese datasets were partioned by hierarchical clustering and the identified groups of correlated genes and miRNAs were validated on Caucasian dataset. The number of genes (C) and miRNAs (D) included in each cluster pair was conserved.

Variability in specimen collection, storage, processing and analysis represents a major source of bias in translational research, producing artifacts and misinterpretation of high-throughput results. Accordingly, all specimens initially collected in Shanghai and Milan were subsequently stored, randomly processed, and analyzed in identical experimental conditions in the Italian center to minimize preanalytical, instrumental, and computational variability between the two series, enabling direct comparison of the gene and miRNA profiles. Agglomerative hierarchical clustering applied to the Chinese–Caucasian dataset validated homogeneity of Chinese and Caucasian expression profiles, since all samples clustered independently of their origin and tended to group according to hormonal receptor status (Fig.1A). Comparable results were observed in clustered miRNA expression profiles of Chinese and Caucasian samples (Fig.1B) and on cluster analysis of selected genes and miRNAs with high variation across samples (IQR > 1) (data not shown). The association between storage time before freezing and gene and microRNA expression was assessed in the Italian dataset to verify whether this technical source of variability could affect the molecular comparison of the 2 groups. Forty-seven (0.3%) of 15,929 genes analyzed and 1 (0.1%) microRNA of 848 showed a significant dependence to storage time (*P* < 0.001, Table S1). No overlap was observed between these 47 genes and 484 of 554 human breast signatures retrieved from GeneSigDB collection; the remaining 70 signatures included from 1 to 5 of the storage-associated genes (Table S2). Therefore, the storage time of samples before freezing impacted the expression of a very limited number of genes and miRNAs and the genes were not involved in pathways or biological processes relevant for breast cancer.

To compare the molecular architecture of Chinese and Caucasian transcriptomes, we tested whether similar clusters of correlated genes, presumably cotranscribed and involved in the same biological processes, were identifiable in both datasets. We identified 59 clusters including at least 10 genes with a Pearson correlation greater than or equal to 0.6 in the Chinese profile and confirmed 54 of the clusters (92%) in the Caucasian dataset (Table S3 and Fig.1C). The same approach was applied to miRNA data, where 31 (97%) of 32 clusters identified in the Chinese dataset were validated in the Caucasian dataset (Fig.1D). Because a major pitfall of this approach rests in the higher probability of validation in larger clusters, the size of clusters identified in Chinese and validated in Caucasian sets was examined to verify the conservation of gene number in each cluster pair; a similar number of genes (Pearson’s coefficient = 0.94) and miRNAs (Pearson’s coefficient = 0.95) was included in Chinese and Caucasian clusters. The entire procedure was repeated using Caucasian gene and miRNA datasets to identify clusters validated in the corresponding Chinese datasets, i.e., 43/54 (80%) gene clusters and 24/24 (100%) miRNA clusters (Fig. S1A and B and Table S4), confirming that expression of pivotal transcriptional features in breast cancer is basically alike in Chinese and Caucasian samples. Finally, Chinese and Caucasian profiles were both used as validation datasets for gene and miRNA clusters identified in an independent dataset which integrated both gene (GSE22219) and miRNA (GSE22216) expression data from 207 samples of primary breast tumor samples from Caucasian English patients profiled on the Illumina platform. A quota of variability derived by different annotation and global gene number of the independent dataset affected the comparison at the gene level, and 53% of gene and 82% of miRNA clusters, identified in the independent dataset were validated both in Chinese and Caucasian Italian datasets (Table S5 and Fig. S2A and B). Functional characterization of Chinese and Caucasian gene-clusters, by testing whether gene lists relevant to breast cancer 1,20–22 were over-represented, revealed a number of clusters of correlated genes in both Chinese and Caucasian datasets that were associated with: (1) intrinsic classification 1,20 defining luminal epithelial/ER and ERBB2 clusters and clusters of genes involved in proliferation and epithelial differentiation; (2) ECM components, including structural and adhesion molecules 21; and (3) immunological mechanisms according to Rody et al. 22, represented by hemopoietic cell kinase (HCK), lymphocyte-specific kinase (LCK), major histocompatibility complex (MHC) and interferon clusters (Table S6).

### Subtyping Chinese and Caucasian samples by intrinsic genes

**Figure 2:**
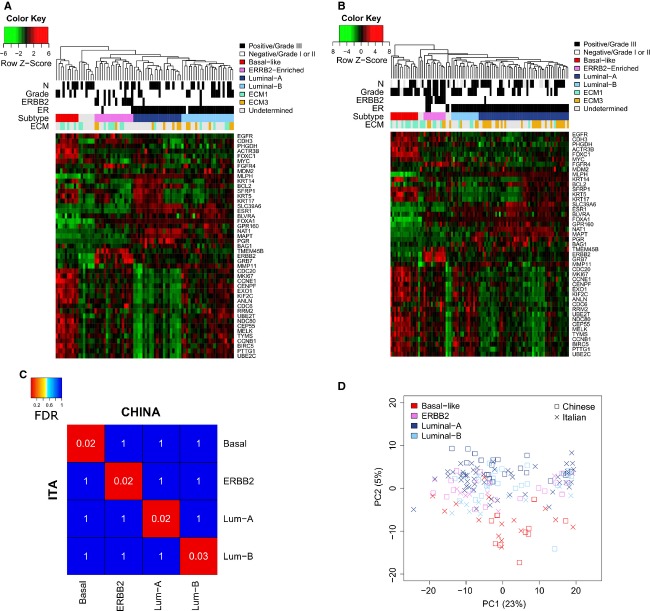
Intrinsic classification of Chinese and Caucasian breast tumors. Unsupervised hierarchical clustering of Chinese (A) and Caucasian (B) samples on the PAM50 intrinsic gene list assigned samples to four breast intrinsic subtypes. Unclassified samples are indicated in gray. Color bars show lymph nodal status (N), histological grade, ERBB2, and estrogen receptor (ER) positivity evaluated according to gene expression level, intrinsic subtype assignment by PAM50, and extracellular matrix (ECM) subtype assignment by the Large Average Submatricies (LAS) biclustering algorithm. (C) Heat map of the subclass association matrix obtained by Subclass Mapping. Gene profiles of intrinsic subtypes identified in the Chinese dataset were nearly identical to those of corresponding subtypes defined in the Caucasian dataset. False discovery rate (FDR) (white) represents the significance of the similarity. ITA and CHINA indicate Caucasian Italian and Chinese samples, respectively. (D) Principal component analysis (PCA) of miRNA expression data in Chinese and Caucasian samples. Grouping of Chinese and Caucasian samples was more associated with intrinsic subtypes than to race/ethnicity.

Chinese and Caucasian samples were assigned to breast cancer intrinsic subtypes by unsupervised hierarchical clustering using the list of 50 intrinsic genes from Parker et al. 24 (Fig.2A and B). None of the 47 genes affected by the storage time before freezing was included in the PAM50 signature (Table S2). Six Caucasian and seven Chinese samples remained unclassified by PAM50 analysis (Fig.2A and B), but analysis of expression profiles of genes characterizing the claudin-low subgroup of basal-like tumors 25 suggested that nine unclassified samples were indeed claudin-low tumors (Fig. S3). Subclass mapping, a method that statistically measures the similarity of predetermined subtypes in independent datasets, was used to test the correspondence of intrinsic molecular subtypes observed in Chinese and Caucasian gene expression data. The heat map of the subclass association matrix from Chinese and Caucasian intrinsic subtypes revealed near-identity of the subgroups identified in both datasets (Fig.2C). Finally, PCA of global miRNA transcriptomes showed that like gene expression, miRNA profiles tended to group Chinese and Caucasian samples according to molecular subtypes, with a more significant separation of both Chinese and Caucasian basal-like samples independent of ethnic origin (Fig.2D).

The prevalence of intrinsic subtypes in Chinese and Caucasian series differed significantly (*P* = 0.006, Table3 and Fig. S4), due mainly to an imbalance in luminal A and luminal B samples, which are characterized by lower and higher expression of proliferation genes, respectively. In the Caucasian Italian dataset, 67% of samples were assigned to the luminal group (51% luminal A and 16% luminal B), while 15% and 12% were classified as basal-like and ERBB2 subtypes, respectively. These proportions were consistent with the intrinsic classification of the Caucasian English tumors from dataset GSE22219 (Fig. S4, *P* = 0.525). In the Shanghai series, 13% of tumors were classified as basal-like and 22% as ERBB2, while luminal tumors represented 56% (27% luminal A and 29% luminal B). To validate the lower prevalence of luminal A subtype observed in our Chinese series, samples profiled in two public datasets of Chinese consecutive primary breast cancer patients were assigned to PAM50 intrinsic subtypes (Table3). GSE48390 dataset included 81 samples from Taiwanese Chinese Han patients and GSE36772 included 100 samples from Singapore, where the population is constituted by 75% of Chinese. Accordingly to Shanghai series, breast tumors from Taiwan and Singapore showed a significant difference in the frequency of intrinsic subtypes in comparison to Caucasian cohorts, with higher disparity in luminal subtypes (Fig. S4, *P* range: 0.00001–0.056). The ratio between luminal A and luminal B was 0.93, 0.44 and 0.66 for Shanghai, Taiwan and Singapore patients, respectively, whereas it was 3.19 for Caucasian Italian and 2.16 for Caucasian English patients (GSE22219, Table3). Comparison of intrinsic subtypes frequency among the three Chinese series revealed no significant differences (Fig. S4, *P* range: 0.284–0.714).

**Table 3:** Frequency of PAM50 intrinsic subtypes in independent series of primary breast cancer from Caucasian and Chinese patients

	Caucasian series		Chinese series		
Number of samples	97	207	78	81	100
Sample origin	Milan	Oxford	Shanghai	Taiwan	Singapore
GEO identifier	GSE59590	GSE22219	GSE59590	GSE48390	GSE36722
PAM50 intrinsic subtype 24 (%)					
Basal-like	15 (15)	34 (16)	10 (13)	8 (10)	8 (8)
ERBB2	12 (12)	32 (15)	17 (22)	15 (19)	17 (17)
Luminal A	49 (51)	88 (41)	21 (27)	15 (19)	25 (25)
Luminal B	15 (16)	42 (19)	23 (29)	35 (43)	38 (38)
Undetermined	6 (6)	20 (9)	7 (9)	8 (10)	12 (12)
Luminal A/Luminal B ratio	3.19	2.16	0.93	0.44	0.66

GEO, Gene Expression Omnibus.

### Subtyping Chinese and Caucasian samples by ECM genes

**Figure 3:**
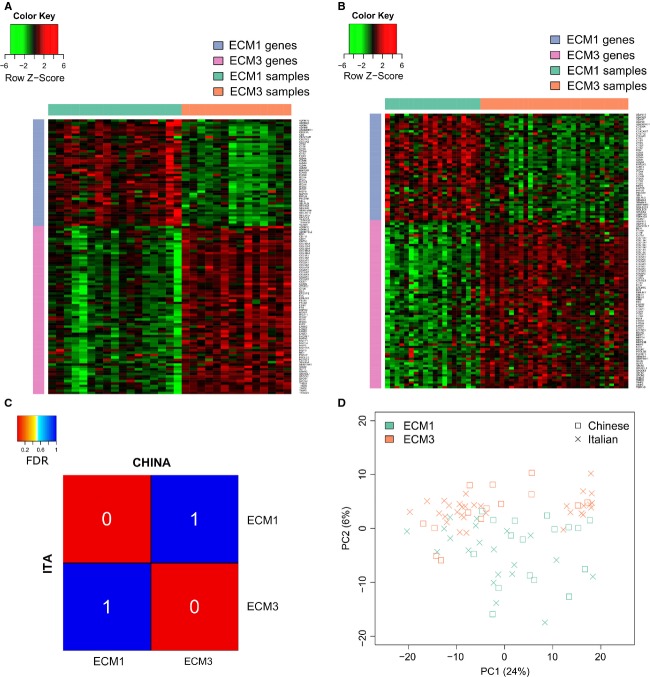
ECM3 and ECM1 subtyping in Chinese and Caucasian breast tumors. Heat map shows the expression profile of ECM3 and ECM1 genes in Chinese (A) and Caucasian (B) tumors identified by LAS biclustering. (C) Heat map of the subclass association matrix obtained by SubClass Mapping revealed nearly identical gene expression of ECM subtypes identified in the Chinese and the Caucasian datasets. FDR (white) represents the significance of the similarity. ITA and CHINA indicate Caucasian Italian and Chinese samples, respectively. (D) PCA analysis of microRNA (miRNA) expression data in Chinese and Caucasian ECM3 and ECM1 breast tumors indicates the greater role of ECM classification than of race/ethnicity in grouping of Chinese and Caucasian samples. ECM, extracellular matrix; PCA, principal component analysis; FDR, false discovery rate; LAS, Large Average Submatricies.

Differential expression of ECM-related genes identified distinct subgroups with different clinical outcome in breast carcinoma 21. LAS biclustering of 738 ECM-related genes identified Chinese and Caucasian ECM3 clusters (Fig.3A and B), whose gene content and stability parameters (Tables S7 and S8) were comparable to those of ECM3 clusters described in 6 independent datasets 26, confirming ECM3 as the most robust ECM cluster in Caucasian and Chinese breast cancers. Likewise, LAS biclustering identified ECM1 clusters with a similar gene pattern and stability in both Chinese and Italian datasets (Fig.3A and B, Tables S7 and S9). Consistent with our previous studies 21,26, the most significant features in determining ECM3 clusters in both Chinese and Caucasian samples were *SPARC*, *BGN*, *CDH11*, *FN1*, *LAMA4*, *MMP2* and several collagens (*COL1A1*, *COL1A2*, *COL5A1*, *COL5A2*, *COL5A3*, *COL6A1*, *COL6A2*, *COL6A3*), whereas ECM1 clusters were characterized by genes encoding cell adhesion molecules (*SELL*, *ITGA4*, *ITGAL*, *ITGAX*, *ITGB2*, *ITGB7*) and enzymes such as granzymes (*GZMA*, *GZMB*, *GZMK*, *GZMH*, *GZMM*), metallopeptidases (*ADAM7* and *ADAM28*) and cathepsins (*CTSC*, *CTSH*, *CTSS*, *CTSW*, *CTSZ*). Subclass mapping revealed extensive similarity in the global gene profiles of ECM clusters identified in the Chinese and Caucasian datasets (Fig.3C), and PCA of miRNA profiles (Fig.3d) supported the transcriptional similarity of ECM3 and ECM1 Chinese and Caucasian tumors.

Prevalence in Chinese and Caucasian series of ECM1 (22% and 21%, respectively) and ECM3 (18% and 32%, respectively) subtypes was not significantly different (*P* = 0.25). Chinese and Caucasian ECM3 tumors were mainly ER-positive (78% and 83%, respectively) while Chinese and Caucasian ECM1 tumors were principally ER-negative (70% and 75%, respectively).

### Differential expression of genes and miRNAs among Chinese and Caucasian samples

**Figure 4:**
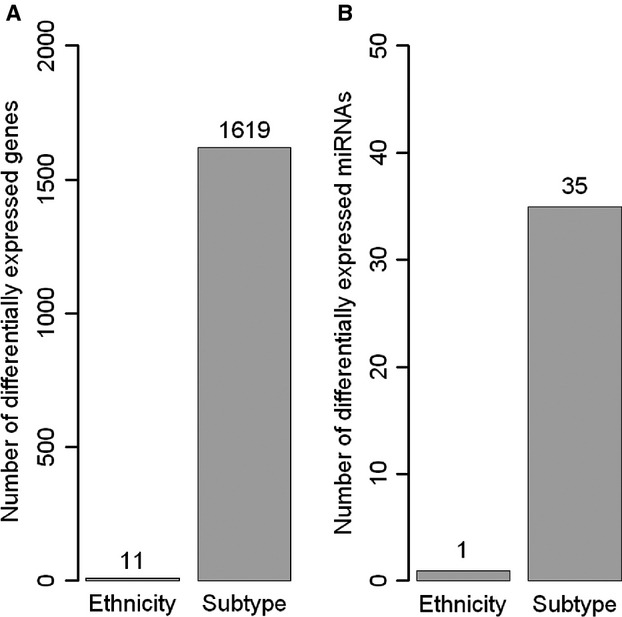
Effect of race/ethnicity and subtypes on gene and microRNA (miRNA) expression. The bar plot reports the number of differentially expressed genes (A) and miRNAs (B) identified by comparison of Chinese and Caucasian samples or by pairwise comparison between subtypes. The number of significant features was dramatically higher when subtypes were compared.

To quantify differences between Chinese and Caucasian samples, differentially expressed genes and miRNAs were identified through direct comparison between all samples followed by comparison among intrinsic subtypes. The number of differentially expressed genes and miRNAs (absolute fold-change ≥2 and FDR < 0.05) according to the molecular subtype significantly outnumbered the differentially expressed features between Chinese and Caucasian samples (Fig.4), suggesting that most of the variability in Chinese and Caucasian expression data reflects biological differences associated with molecular subtype rather than ethnic origin. Moreover, one of the 11 genes and the only miRNA differentially expressed between Chinese and Caucasian samples were significantly affected by storage time before freezing, indicating that a fraction of the observed differences derived from technical factors.

## Discussion

We find here that comprehensive molecular portraits of breast cancer transcriptomes originating from gene and miRNA expression of Chinese Han and Caucasian Italian subjects are remarkably similar. We therefore provide evidence that intrinsic classification of Chinese breast cancer, as carried out previously in Asian cohorts 30,31, is indeed equivalent to Caucasian breast tumors, since equal entities were identified in Chinese and Caucasian cohorts classified by PAM50.

We took advantage of a unique collection of breast cancer frozen specimens from Shanghai Fudan Cancer Center profiled simultaneously with an analogous Caucasian Italian series that yielded transcriptomic data devoid of batch effects. Gene and miRNA profiles of Chinese and Caucasian samples were compared using an unsupervised approach that highlighted similar clusters of correlated features in Chinese and Caucasian samples of our study and in an independent dataset of Caucasian patients, indicating that the taxonomy of transcriptional elements regulating breast cancer biology was the same in the Chinese and Caucasian groups. Interestingly, the transcriptomic diversity between the Caucasian Italian and English groups profiled in different hospitals seemed greater than that between the Chinese and Caucasian groups profiled simultaneously on the same platform.

Partition of gene expression data using predetermined gene lists including “intrinsic” or “ECM” genes identified Chinese and Caucasian subgroups with equivalent gene and miRNA profiles. Thus, breast tumors in the Chinese and Caucasian groups appear to be subjected to the same molecular and differentiation processes. Similar transcriptional profiles and cluster characteristics of Chinese and Caucasian ECM3 and ECM1 tumors suggest similarity in the tumor-surrounding stroma characteristics, including tumor infiltration, since the expression of genes related to immunological processes 22 was also consistent between Chinese and Caucasian samples. Finally, although the miRNA breast cancer transcriptome is less well-defined, we found an overall transcriptional similarity of Chinese and Caucasian miRNA patterns, indicating that the main control circuitries regulating gene expression are conserved in Chinese breast cancer. Nevertheless, we cannot exclude the possibility that some genes or miRNAs differentially expressed among Chinese and Caucasian samples have a slight functional effect or that functional traits not associated with transcriptional changes, such as protein modifications or some metabolic pathways, might differ.

Although intrinsic subtypes of the Chinese and Caucasian series were biologically similar, their prevalence differed significantly, with a reduced fraction of luminal A tumors in Chinese samples from Shanghai. This finding, that was validated in 2 independent series of Chinese women from Taiwan and Singapore, might indicate an actual difference in the prevalence of this luminal tumor subgroup in the Chinese series, as also suggested by our findings of a significant disparity in prevalence of ER-positive disease in a large cohort of Chinese and Caucasian patients from Fudan and INT, respectively. Consistent with our data, a study of breast cancer patients in Shanghai 32 and a multicenter analysis of breast cancer patients from 7 distinct hospitals across China 33 reported 65% and 57% ER-positive tumors, respectively. The younger age of tumor onset in Chinese series might explain the reduced prevalence of luminal tumors compared to Caucasians, considering that age and ER status were associated with Chinese women in our study. This assumption is supported by the recent continuous and simultaneous increase of age of tumor onset and prevalence of ER positivity of breast cancer in China 8. Rapid lifestyle changes that Chinese women experienced in the last decades, including dietary patterns and reproductive factors that are well known to influence the hormonal profile, and the biological similarity of breast cancer from Caucasian and Chinese women, point to the likelihood that the incidence of low-grade ER-positive tumors in postmenopausal Chinese women will increase to levels currently seen in Caucasian breast cancer 34. Further large comparative studies of Asian and Caucasian cohorts homogeneous for geographic origin and socioeconomic status are needed to fully understand the significance of age or other risk factors in the prevalence of ER- positive disease in China.

The substantial similarity of breast cancer transcriptomes from our different ethnic groups is in agreement with findings in comparative analyses of Afro-American and Caucasian women with breast cancer, in whom similarity at the transcriptional and protein levels 35 and similar DNA methylation status of 7/8 frequently hypermethylated genes in breast tumor tissues 36 were found. Nevertheless, epidemiological differences in breast cancer incidence/mortality and strikingly distinct somatic mutation spectra between subjects and groups have been reported 6, indicating that cancer diversity across ethnic populations is complex and not well understood. Complexity in collecting homogeneous and comparable data in multiethnic cohorts and small sample size, study design and socioeconomic conditions, in addition to heterogeneity of breast cancer disease, all affect transethnic studies. A paradigmatic example is the reportedly increased mortality of Afro-American women partially due to socioeconomic disparity but also associated with the prevalence of the most aggressive TNBC subtype in premenopausal women 37; upon subtype-specific analysis, differences in mortality risk among Afro-American and Caucasian women with TNBC disease vanished 38.

We lacked information on the mutational landscape of our Chinese cohort, and several reports on breast cancer in Asian women indicated both mutational similarity 39 and variations in comparison to Caucasian women 40–43. It is well-recognized that a complex pattern of somatic mutations involving thousands of genetic entities in cancer of single individuals 4,5 indeed merges in a Darwinian way to a restricted number of functional alterations common to cancer of different tissues 44. In this context, our data suggest that although the somatic mutational landscape of breast cancer in Chinese and Caucasian patients might differ, the resulting transcriptomes of both groups retain a similar global architecture distinctive of breast cancer, leading to equivalent functional relationships. This finding has ramifications for translational medicine and personalized approaches since similarity of breast cancer biology across ethnic groups will influence clinical management of patients worldwide and facilitate development of anti-cancer drugs.

## Supporting Information

**Figure S1.** Number of genes (A) and miRNAs (B) included in each cluster identified in the Caucasian Italian dataset and validated in the Chinese dataset. Chinese and Caucasian cluster size showed a Pearson correlation of 0.96 and 0.87 for genes and miRNAs, respectively. **Figure S2.** Number of genes (A) and miRNAs (B) included in each cluster identified in GSE22219 and validated in both Caucasian Italian and Chinese datasets. For genes, size of cluster pairs showed a Pearson correlation of 0.77 and 0.79 for GSE22219 versus the Chinese dataset and versus the Italian dataset, respectively; for miRNAs, size of cluster pairs showed a Pearson correlation of 0.71 and 0.76 for GSE22216 versus the Chinese dataset and versus the Italian dataset, respectively. **Figure S3.** Unsupervised hierarchical clustering of merged Chinese and Caucasian Italian samples using the claudin-low gene list. Nine of the thirteen Chinese and Caucasian samples designated as unclassified by the PAM50 signature were identified as claudin-low tumors. **Figure S4.** Square-matrix reporting the *P*-values obtained from the comparison of PAM50 intrinsic subtypes frequencies between each pair of Chinese and Caucasian group. Colors represent the strength of the significance: red, significant; green, not significant.

**Table S1.** List of genes significantly associated with the storage time before freezing. **Table S2.** Overlap between genes associated with the storage time before freezing and genes included in the breast cancer signatures retrieved from GeneSigDB database. **Table S3.** Genes included in clusters of correlated genes identified in the Chinese dataset and validated in the Caucasian Italian dataset. The value 0 indicates that the gene was not included in the gene cluster in the validation phase. **Table S4.** Genes included in clusters of correlated genes identified in the Caucasian Italian dataset and validated in the Chinese dataset. The value 0 indicates that the gene was not included in the gene cluster in the validation phase. **Table S5.** Genes included in clusters of correlated genes identified in the public Caucasian dataset GSE22219 and validated in our Chinese and Caucasian Italian datasets. The value 0 indicates that the gene was not included in the gene cluster in the validation phase. **Table S6.** Enrichment of relevant breast cancer biological signatures in gene-clusters identified in Chinese, Caucasian Italian, and Caucasian English datasets. **Table S7.** List of genes defining ECM1 and ECM3 subtypes in Chinese and Caucasian Italian datasets. **Table S8.** Stability analysis of ECM3 clusters. Statistical methods used to compare Chinese and Caucasian ECM clusters are detailed in the supplementary material of Triulzi et al. 26. **Table S9.** Stability analysis of and ECM1 clusters. Statistical methods used to compare Chinese and Caucasian ECM clusters are detailed in the supplementary material of Triulzi et al. 26.
