# Supplementary material for: Molecular portrait of breast cancer in China reveals comprehensive transcriptomic likeness to Caucasian breast cancer and low prevalence of luminal A subtype
Source: Cancer Med. 2015 Mar 18;4(7):1016–30. doi: 10.1002/cam4.442 (PMC4529340; doi:10.1002/cam4.442)
Supplement: Supplementary file 1 [file cam40004-1016-sd1.pptx]

## Slide 1
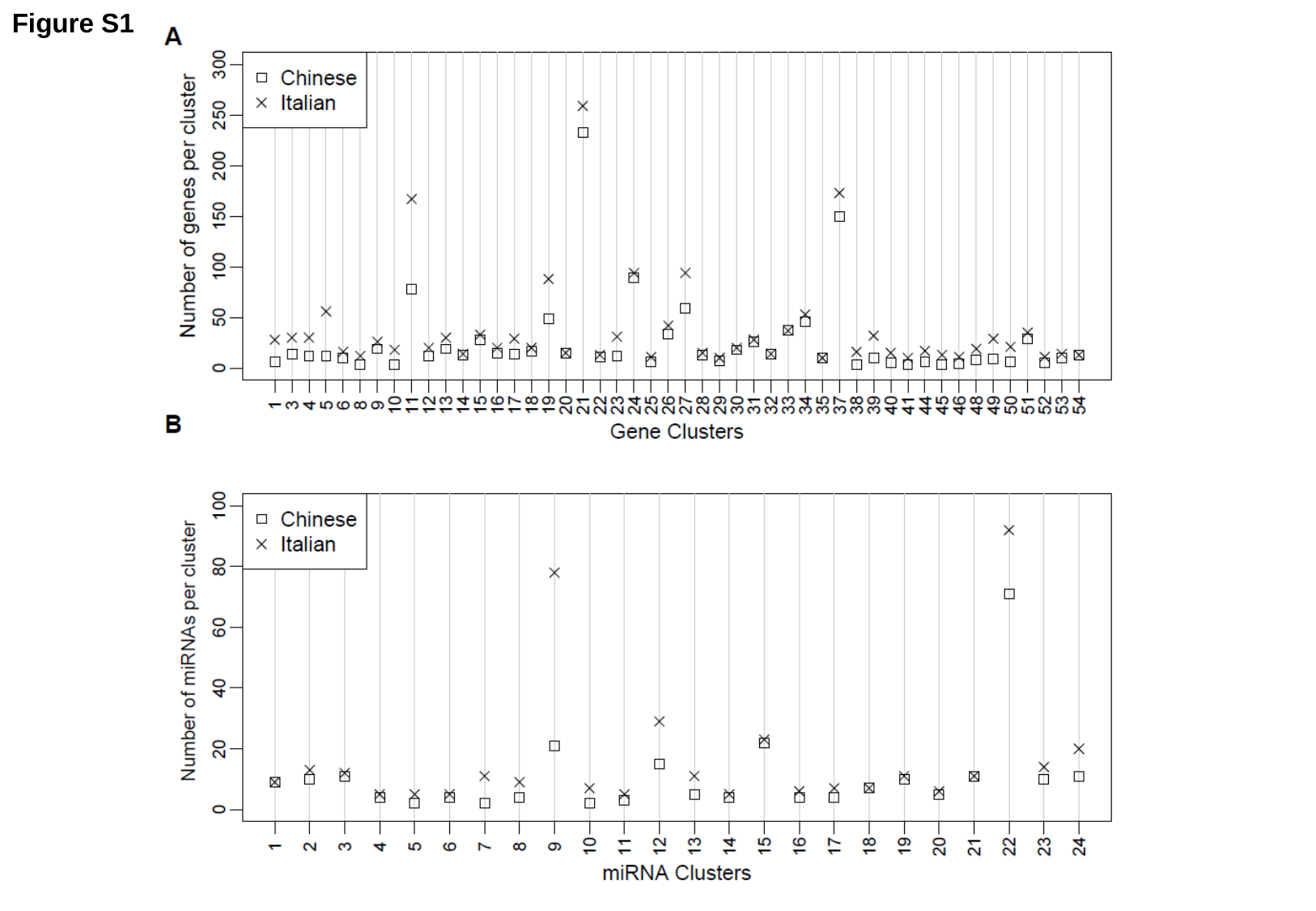

Figure S1

## Slide 2
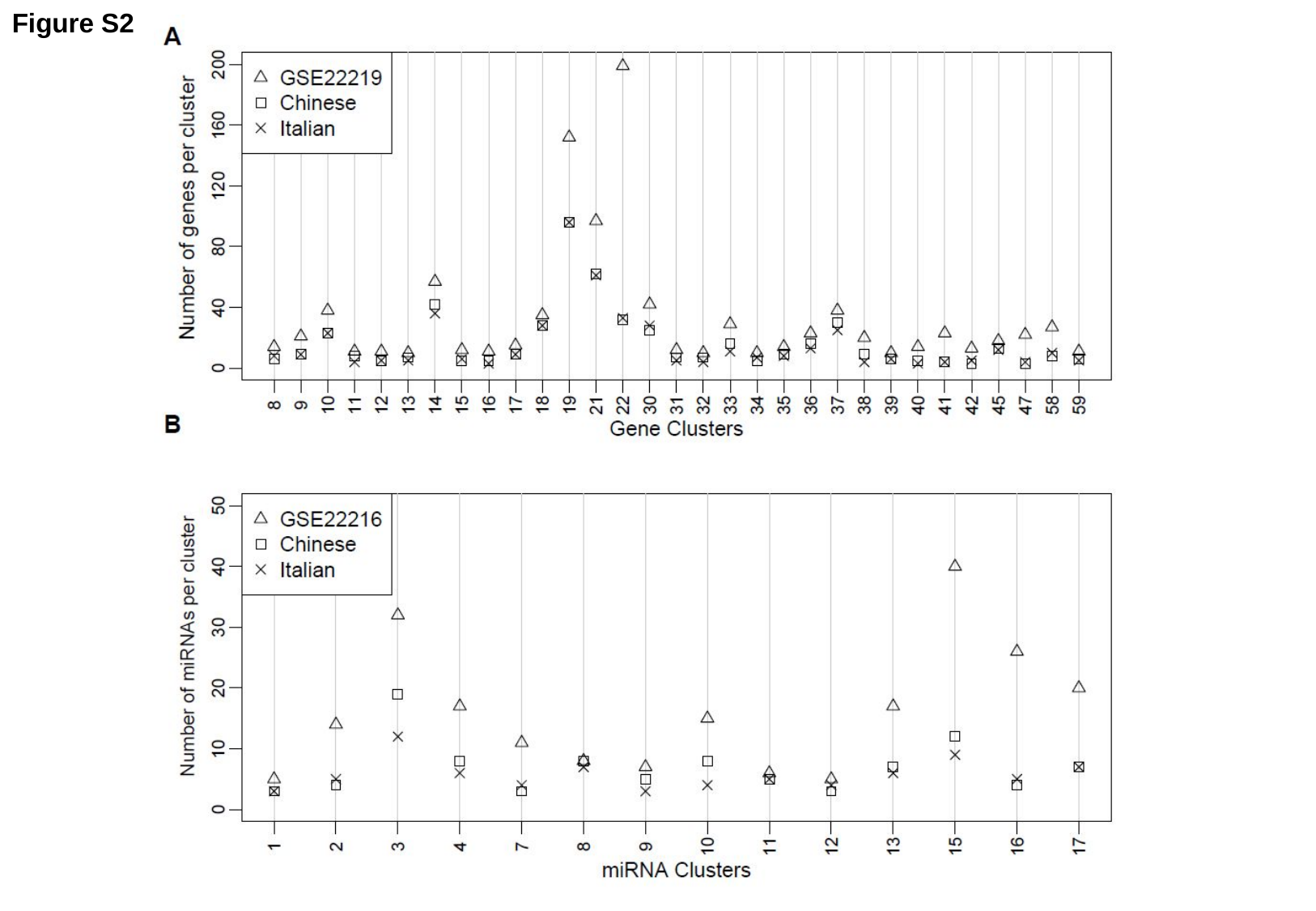

Figure S2

## Slide 3
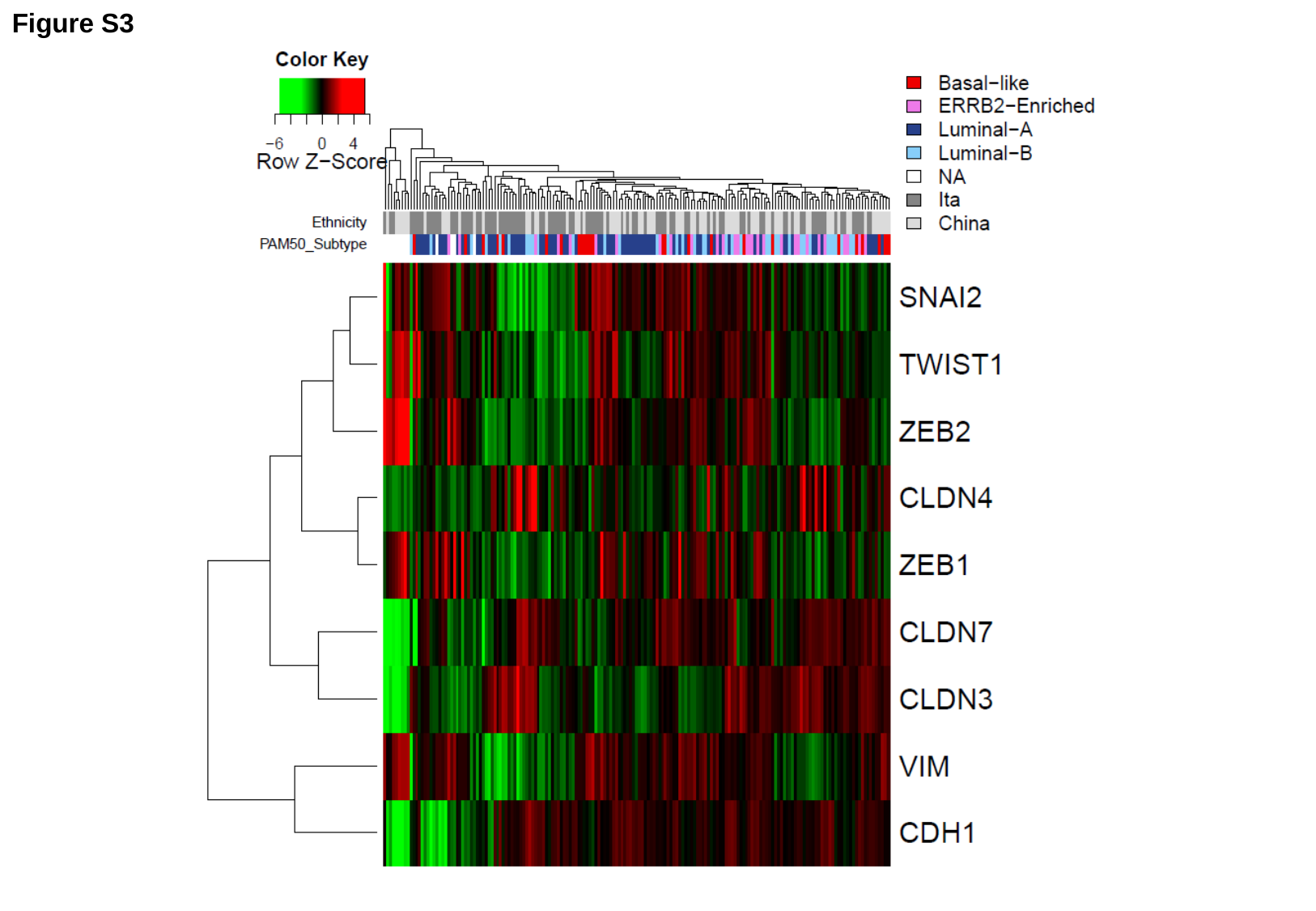

Figure S3

## Slide 4
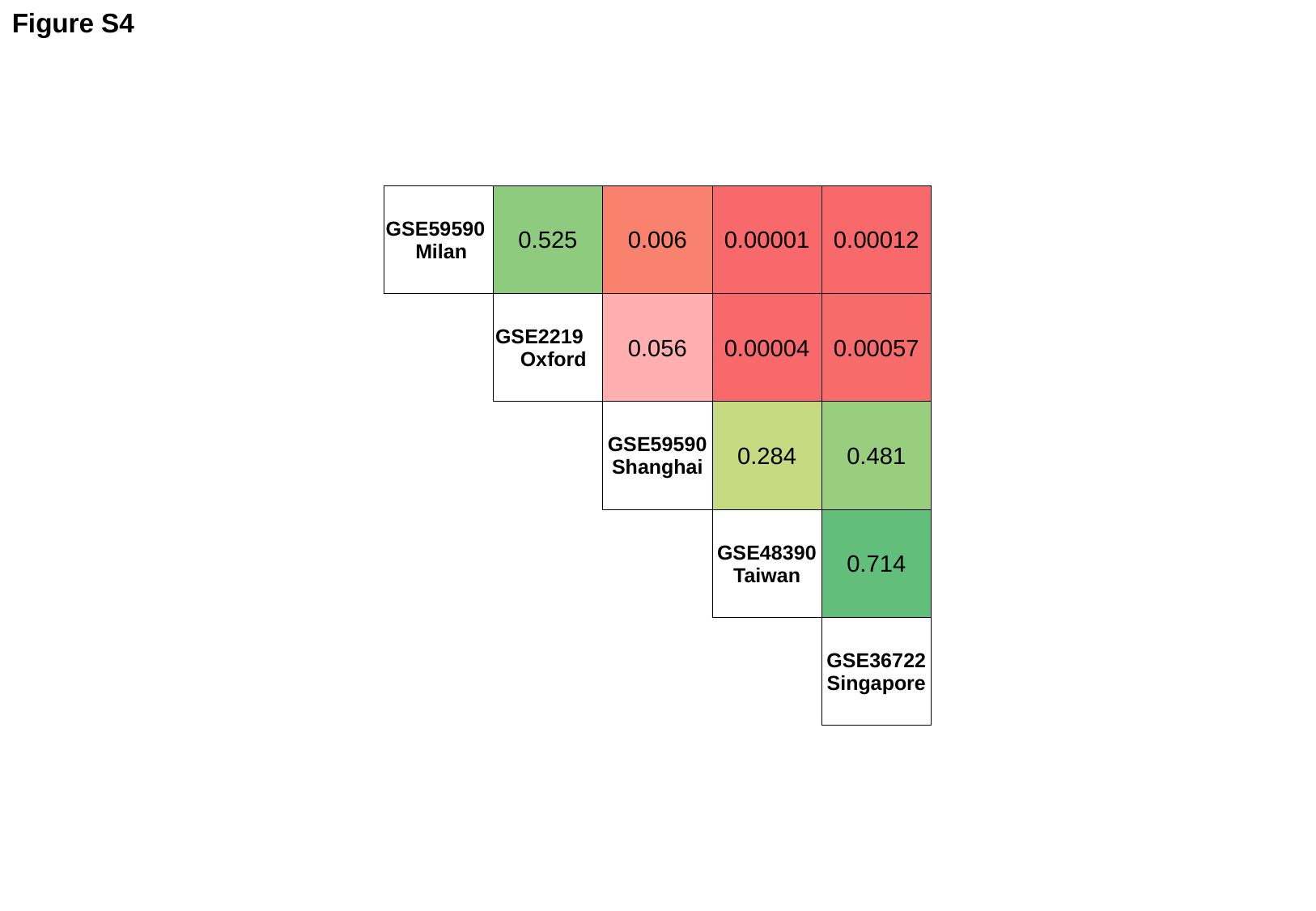

Figure S4
| GSE59590 Milan | 0.525 | 0.006 | 0.00001 | 0.00012 |
| --- | --- | --- | --- | --- |
| | GSE2219 Oxford | 0.056 | 0.00004 | 0.00057 |
| | | GSE59590 Shanghai | 0.284 | 0.481 |
| | | | GSE48390 Taiwan | 0.714 |
| | | | | GSE36722 Singapore |
